# Social determinants of the non-use of the explicit health guarantees plan (the GES plan)

**DOI:** 10.1186/s12913-023-10149-8

**Published:** 2023-10-19

**Authors:** Sandra Alvear-Vega, Héctor Vargas-Garrido

**Affiliations:** 1 Faculty of Business and Economics, University of Talca, Talca, Chile; 2 Faculty of Psychology, University of Talca, Talca, Chile

**Keywords:** Universal health coverage (UHC), Explicit Health guarantees (GES), Social determinants, Vulnerable health groups

## Abstract

**Introduction:**

The public policy called Explicit health guarantees (GES) could serve as a basis for the future implementation of universal health coverage in Chile. An improvement in the quality of health of the Chilean population has been observed since the launching of the GES, which has a high adherence (84% of the beneficiary population uses this health program). This work seeks the social determinants related to a portion of the remaining 16% of people who do not use the GES.

**Methods:**

This secondary analysis study used a sample of GES recipients (*n* = 164,786) from the National Socioeconomic Characterization Survey (CASEN) 2020. The GES recipients included in the study responded that they had been under medical treatment for 20 of the 85 pathologies included in the GES, and they had not had access to such policy due to “trust in physician/facility,“ “decided not to wait,“ or “lack of information.” The CASEN survey chose the 20 pathologies. The Average Marginal Effects of social determinants of the non-use of the GES health plan were predicted using multivariable and panel multinomial probit regression analyses, where the outcome variable assumed three possible values (the three reasons for not accessing) while taking those variables reported in previous studies as independent variables.

**Results:**

A higher probability of non-access due to distrust in the physician/facility among adults with higher economic income was found. Among those who prefer not to wait are vulnerable groups of people: women, people with a lower-middle income, those who belong to groups with longer waiting times, and ethnic groups. The people who least access the GES due to lack of information correspond to part of the migrant population and those belonging to the lowest income group.

**Conclusions:**

The GES policy must necessarily improve the timeliness and quality of the services to make them attractive to groups that currently do not have access to them, managing waiting times rather than referrals and using patient-centered evaluations, especially in those most vulnerable groups that do not access GES because they choose not to wait or lack the necessary information, thereby improving their health literacy.

## Background

Universal health coverage (UHC) is a public policy promoted by the United Nations that seeks to ensure that all people have equitable access to quality health services, when and where they need them, without financial hardship. The objective is to cover the full range of health services throughout life, from promotion to prevention, treatment, rehabilitation, and necessary palliative care [1]. Each country has adopted its model of universal health coverage, ranging from preferential state participation (as is the case in the United Kingdom) to mixed models of public and private participation (such as in Switzerland, Germany, and the Netherlands) [2]. Universal health coverage seeks to protect people financially in the short term [1], and in the long term, it is oriented towards lower health spendings for countries and obtaining the social benefits derived from a healthier population [3, 4]. However, this model is not free of questions. On the one hand, in terms of health care offering, there are objections due to the high costs involved in infrastructure, the coverage of people without previous health insurance, and the expansion of the provision of health services, among others; on the other hand, considering health demand, there are objections in that this model does not consider the diverse health needs of some populations due to climatic heterogeneities, housing, and cultural densities, as well as long waiting times for patients, among others [4].

With the application of the UHC policy, a key challenge observed in different countries is to incorporate equity criteria among the healthcare demanders; financial and non-financial barriers place some groups in a disadvantaged or vulnerable position [5–11]. Among the non-financial barriers, the World Health Organization (WHO) particularly pointed out that lower-income groups and rural communities might be disadvantaged [11]. Research also emphasizes the need to consider these important factors [5–10]. For example, in a systematic review conducted in African countries, Sanogo and colleagues [5] found that some factors that conditioned access to and use of health services, under the universal access modality, were sociocultural barriers, physical inaccessibility, and lack of education and information, among others. Likewise, in South Korea, the percentage of people affected by non-financial barriers is higher than those affected by financial barriers, where women and people with functional limitations stand out among the most vulnerable groups [7]. Similarly, other studies warn about the vulnerabilities associated with living in marginal and informal neighborhoods [9] and among the migrant population [10] related to participation in the UHC. In the Latin American context, these countries segregate the population into two groups within the health system: a suitable social security system for enough-income workers and a public system that serves the least favored and vulnerable people. This segregation has become a barrier to implementing the right to universal health care as it perpetuates the economic, social, and health inequality that characterizes Latin America [12, 13]. For this reason, some countries have implemented measures to address the social determinants that generate inequalities; for example, poverty alleviation, support for disadvantaged children, and development of education and employability programs, among others [13]. Thus, studying social determinants becomes especially relevant to implement strategies that correct the challenges and ultimately contribute to the efficient implementation of UHC policies [5].

In this context, Chile has implemented a model called Explicit Health Guarantees (GES) (Law 19.666/2004). This health policy establishes the set of benefits (promotional, preventive, curative, rehabilitative, and palliative) that both the public provider (National Health Fund; called FONASA) and private providers (Social Security Health Institutions, called ISAPRES) must cover for their respective beneficiary populations. The guarantees established by law are related to the access (obligation of public and private entities to provide the service), quality (certified and accredited health care services), financial protection (predetermined co-payments), and timeliness of care (deadlines for delivery of services) [14]. The above characteristics have led some authors to suggest that the current GES plan could serve as a basis for the future implementation of universal health coverage in the country [15]. Until 2020, the GES plan covered 85 pathologies [14], with waiting times being one of the main limitations in its implementation [16]. Together with the implementation of GES, the Chilean health system has provided broader responses to the country’s health needs, although it still presents fundamental challenges related to access barriers [17, 18]. A recent work found that out of the total medical care entitled to be covered by the GES plan, in 16% of the cases, the affected individuals did not use this public health policy [15]. Therefore, the purpose of this study is to identify the social determinants related to those beneficiaries, both in the Chilean public and private systems, who do not use the GES to obtain helpful information for the improvement of public health programs. We will work with the data provided by CASEN 2020 [19].

## Materials and methods

### Source of information

The data for this quantitative secondary analysis study were obtained from the National Socioeconomic Characterization Survey, CASEN 2020, carried out by the Surveys and Longitudinal Studies Center of the Catholic University of Chile (PUC). The sample units of the Casen 2020 survey are households selected in a probabilistic, stratified, and multistage manner. Within the dwelling, all family units and persons who are members of each one are identified. Interviewers applied a paper-and-pencil survey in a face-to-face procedure. The GES recipients included in the study responded that they had been under medical treatment for 20 of the 85 pathologies included in the GES (the CASEN survey chose these 20 pathologies) and provided information regarding the following issue: “Why this medical treatment was not covered by the AUGE-GES system (S30).“ Thus, the data for this study comprised a sample of 1,446 people who answered the last question due to “trust in physician/facility,“ “decided not to wait,“ or “lack of information,” using the expansion factor (expr) to reach 164,786 individuals. The regional expander (expr) allows us to obtain results of individuals and family units expanded at national, regional, and area levels (urban and rural), being representative of the country’s total population [19]. All interviewees voluntarily participated, and anonymity was guaranteed according to Law 17.374. The CASEN is of public use and is used in research as a secondary data source [20].

### Outcome variable: “Trust in physician/facility,“ “Decided not to wait,“ and “Lack of information”

In response to the CASEN 2020’s question: “Why this medical treatment was not covered by the AUGE-GES system?”, respondents had nine response options (excluding those associated with COVID-19), which are indicated by their frequency percentages: (1) “You preferred to choose another physician or facility or to continue with your usual physician” (47%); (2) “You decided not to wait to access care through AUGE-GES, to solve your problem more quickly” (14%); (3) “You thought that AUGE-GES care could be of low quality” (3%); (4) “Your health insurance covered your need better than AUGE-GES” (6%); (5) “The procedure to access AUGE-GES is complicated” (5%): (6) “AUGE-GES did not cover the needs of your medical condition” (9%): (7) “I did not know that AUGE-GES covered my condition” (13%); (8) “I do not belong to the age group covered by AUGE-GES” (2%); and (9) “Your usual physician advised you not to seek care under AUGE-GES” (1%). Based on that information, this research poses the following question: Which social determinants relate to non-access to the GES plan? In defining the outcome variable, the nine response options were grouped, according to Tanahashi’s model [21], into three categories: acceptability (items 1, 3, and 9), accessibility (items 2, 4, and 5), and availability (items 6, 7, and 8). However, the probit model detected collinearity among them, which precluded the development of statistical analyses. Thus, we decided to work only with the three choices that had the highest frequency of responses: “You preferred to choose another physician or facility or to continue with your usual physician” (*n* = 107,643; 65%); 2) “You decided not to wait to access care through AUGE-GES to solve your problem more quickly” (*n* = 33,052; 20%); and 3) “I did not know that AUGE-GES covered my condition” (*n* = 24,091; 15%). For simplicity, the following labeling of the variables were used: “trust in physician/facility,“ “decided not to wait,“ and “lack of information,“ respectively. Thus, the dependent variable is the nominal categorical, with three response options (*N* = 164,786).

### Independent variables

The independent variables were grouped following classifications from previous studies [15, 20, 22]. Out of the seven thematic modules of the CASEN 2020 (registration-residents, education, work, income, health, identities, and housing), three dimensions were grouped: (a) demographic variables (sex, age, native peoples’ descendants, immigrant, and geographic area); (b) social variables (income quintile, educational level, informal job, professional performance area, public or private mandatory health insurance, supplementary health insurance); (c) illnesses or health variables (all those 20 GES pathologies included in the CASEN survey, such as primary arterial hypertension, diabetes mellitus, depression, among others).

### Plan of analysis

First, a descriptive analysis of the variables was performed. Subsequently, a multinomial probit model was generated to establish the relationship between the three options for non-use of the GES health plan (outcome variable) and the independent variables. For statistical analysis, the Stata14 software was employed. A set of coefficients, namely *β1*, *β2*, and *β3*, representing “trust in physician/facility,” “decided not to wait,” and “lack of information”, were used to analyze data and obtain the Average Marginal Effects (AMEs) [23]. Through this technique, it is possible to measure the impact that, on average, each independent variable (controlling for the remaining factors) has on the probability that a person does not access the GES, corresponding to the following equation:

$$AME=\frac{1}{N}\sum\limits_{{i=1}}^{N} {\frac{{\partial E\left[ {yi/{x_i},{w_i}} \right]}}{{\partial x}}} \beta x$$

### Goodness of fit

The model’s estimation was based on 164,786 observations, and the log-likelihood was − 134,953.17, *χ*^2^ (68) = 20,613.56, *p* < .0001. The goodness of fit, Count R2 = 0.66, indicates good explanatory power. The independent variables explained between 11.7% (Cox-Snell) and 14.1% (Nagelkerke) of the variance of the outcome variable. The model, by default, established “trust in physician/facility” as the reference group; therefore, it estimated a model for non-access to the GES plan due to “decided not to wait” and “lack of information.“

## Results

### Sample description

People who did not access the GES plan due to “trust in physician/facility” (*n* = 107,643) were primarily men (*n* = 54,178; 50.3%), had a public health mandatory insurance (*n* = 56,684; 52.6%), had no supplementary health insurance (*n* = 79,090; 73.4%), were not indigenous peoples’ descendants (*n* = 101,635; 94.4%), were born within the country (*n* = 102,981; 95.6%), had no informal job (*n* = 83,924; 77.9%), and were mostly aged between 36 and 60 years (*n* = 57,719; 53.6%).

Likewise, people who did not access the GES plan because they “decided not to wait” (*n* = 33,052) were mostly women (*n* = 20,129; 60.9%), had public health insurance (*n* = 24,098; 72.9%), had no supplementary health insurance (*n* = 25. 882, 78.3%), were not descendants of indigenous peoples (*n* = 30,180, 91.3%), were born in the country (*n* = 32,798; 99.2%), had no informal job (*n* = 25,104, 75.9%), and were mostly aged between 36 and 60 years (*n* = 11,896; 36%).

Similarly, those individuals who did not access the GES plan due to “lack of information” (*n* = 24,091) were mostly women (*n* = 12,561; 52.1%), had public health insurance (*n* = 16,868; 70.0%), had no supplementary health insurance (*n* = 20. 513, 85.1%), were not descendants of indigenous peoples (*n* = 22,773; 94.5%), were born in the country (*n* = 22,729; 94.4%), had no informal job (*n* = 18,559; 77.0%), and were mostly aged between 36 and 60 years (*n* = 14,050; 58.3%).

### The average marginal effects (AMEs)

The results will be presented following the three dimensions of this study. Variables with statistical significance within the model will be shown.

Regarding demographic variables (see Table 1), according to the model results, women had lower AMEs (-6.0%) than men of not accessing the GES plan due to “trust in physician/facility”; however, women had higher AMEs of not accessing the GES plan due to “decided not to wait” (6.0%).

**Table 1:** Average Marginal Effects (AMEs) of the non-use of the GES health plan, demographic variables

Variable	Values	Predict (Trust in physician/facility)		Predict (Decided not to wait)		Predict (Lack of information)	
		dy/dx	[95%. Conf. Interv.]	dy/dx	[95%. Conf. Interv.]	dy/dx	[95%. Conf. Interv.]
Gender	Female = 1, Male = 0.	− 0.0600***	[-0.0649; − 0.0550]	0.0603***	[0.0561; 0.0645]	− 0.0003	[-0.0041; 0.0034]
Age 0 a ≤ 18 years (base)	(a)						
Age > 18 to ≤ 35 years	(a)	0.1307***	[0.1155; 0.1462]	− 0.0573***	[-0.0705; − 0.0442]	− 0.0734***	[-0.0853; − 0.0615]
Age > 36 to ≤ 60 years	(a)	0.0600***	[0.0460; 0.0739]	0.0082	[-0.0036; 0.0201]	− 0.0682***	[-0.0791; − 0.0574]
Age ≤ 61 years	(a)	0.0785***	[0.0650; 0.0921]	− 0.0480***	[-0.0595; − 0.0365]	− 0.0305***	[-0.0411; − 0.0199]
Native ethnic groups’ descendants	(b)	− 0.0540***	[-0.0632; − 0.0448]	0.0807***	[0.0733; 0.0881]	− 0.0267***	[-0.0339; − 0.0194]
Immigrant	(b)	0.1342***	[0.1195; 0.1488]	− 0.2491***	[-0.2646; − 0.2336]	0.1149***	[0.1062; 0.1235]
Large northern zone	(a)	0.0159	[-0.0076; 0.0395]	0.1023***	[0802; 0.1245]	− 0.1182***	[-0.1346; − 0.1020]
Small northern zone	(a)	0.0039*	[-0.0194; 0.0273]	0.1063***	[0.0844; 0.1283]	− 0.1102***	[-0.1262; − 0.0944]
Metropolitan region	(a)	0.0944***	[0.0888; 0.1001]	− 0.0922***	[-0.0968; − 0.0875]	− 0.0022	[-0.0065; 0.0020]
Central zone	(a)	− 0.1240***	[-0.1462; − 0.1017]	0.1911***	[0.1700; 0.2121]	− 0.0671***	[-0.0819; − 0.0522]
Southern zone	(a)	− 0.0202**	[-0.0426; − 0.0022]	0.0967***	[0.0755; 0.1180]	− 0.0765***	[-0.0915; − 0.0615]
Austral zone (base)	(a)						

Note: dy/dx for factor levels is the discrete change from the base level, which is the margin effect

(a) Inside the range = 1, outside the range = 0; (b) Yes = 1, no = 0

*p < .05; **p < .01; ***p < .001

People in the age group between 19 and 35 years had higher AMEs (13.0%) of not accessing the GES plan due to “trust in physician/facility” than people aged between 0 and 18 years (reference group defined by the model). At the same time, the former had lower AMEs of non-access, with regards to the same age group, due to “decided not to wait” and “lack of information” (-5.7% and − 7.3%, respectively). Likewise, people of the 36 to 60-year bracket had higher AMEs of not accessing the GES plan due to “trust in physician/facility” and “decided not to wait” (6.0% and 0.8%, respectively) than people belonging aged between 0 and 18 years; however, those between 36 and 60 years had lower AMEs of not accessing, concerning the same age bracket, due to “lack of information” (-6.8%). In addition, people older than 61 years had higher AMEs (7.8%) of not accessing the GES plan due to “trust in physician/facility” than people aged between 0 and 18 years. Also, at the same time, they had lower AMEs of not accessing the plan due to waiting lists and lack of information (-4.8% and − 3.0%, respectively) (see Table 1). Likewise, people who identified themselves as of indigenous descent had lower AMEs of not accessing the GES plan due to “trust in physician/facility” and “lack of information” (-5.4% and − 2.6%, respectively) than people who did not. Also, at the same time, they had higher AMEs of not accessing the GES than the same reference group because they “decided not to wait” (8.0%). Likewise, people who were not born in Chile had higher AMEs of not accessing the GES plan due to “trust in physician/facility” and “lack of information” (13.4% and 11.4%, respectively) than people who were born in Chile: also, they had lower AMEs of non-access, compared to the same group, because they “decided not to wait” (-24.9%).

The geographical location displayed that people living in the metropolitan region had higher AMEs of not accessing the GES due to “trust in physician/facility” (9.4%) compared to people living in the Austral zone of Chile. Also, people living in the central, small northern, large northern, and southern zones had higher AMEs of not accessing the GES plan due to waiting lists than those living in the Austral zone of Chile (19.1%, 10.6%, 10.2%, and 9.6%, respectively). People living in the Austral zone of Chile had higher AMEs of not accessing the GES plan due to “lack of information” compared to all the other zones of the country.

Within social variables (Table 2), people with no formal education had lower AMEs of not accessing the GES plan due to “trust in physician/facility” and “lack of information” (-10.4% and − 0.5%, respectively) than people with technical and university education. At the same time, people with no formal education had higher AMEs of non-access, regarding the same group, due to the waiting lists (10.9%) (see Table 1). People with compulsory formal education had higher AMEs of not accessing the GES plan due to “trust in physician/facility” and “lack of information” (1.4% and 1.2%, respectively) than people with technical and university education. In this line, people with compulsory formal education had lower AMEs of non-access, concerning the same reference group, due to the waiting lists (-2.6%). In addition, people with informal jobs had higher AMEs of not accessing the GES plan due to “trust in physician/facility” and “decided not to wait” (0.9% and 2.3%, respectively) than people who had no informal job; at the same time, the former had lower AMEs of non-access, compared to the same group, due to lack of information (-3.2%) (see Table 1).

**Table 2:** Average Marginal Effects (AMEs) of the non-use of the GES health plan, social variables

Variable	Values	Predict (Trust in physician/facility)		Predict (Decided not to wait)		Predict (Lack of information)	
		dy/dx	[95%. Conf. Interv.]	dy/dx	[95%. Conf. Interv.]	dy/dx	[95%. Conf. Interv.]
Without education	(a)	− 0.1044***	[-0.1608; − 0.0480]	0.1094***	[0.0650; 0.1539]	− 0.0050	[-0.0422; 0.0321]
Compulsory education	(a)	0.0145***	[0.0079; 0.0210]	− 0.0269***	[-0.0324; − 0.0214]	0.0124***	[0.0074; 0.0174]
Technical and university education (base)	(a)						
Informal job	(b)	0.0094**	[0.0035; 0.0152]	0.0237 ***	[0.0188; 0.0286]	− 0.0331***	[-0.0376; − 0.0287]
Managers and administrative directors	(b)	0.1696***	[0.1553; 0.1840]	− 0.1090***	[-0.1213; − 0.0967]	− 0.0606***	[-0.0718; − 0.0493]
Professionals, scientists, and intellectuals	(b)	0.1442***	[0.1335; 0.1549]	− 0.0759***	[0.1347 0.1550]	− 0.0683***	[-0.0765; − 0.0602]
Technicians and middle professionals	(b)	0.1155***	[0.1049; 0.1262]	− 0.0675***	[-0.0763; − 0.0586]	− 0.0480***	[-0.0560: − 0.0400]
Administrative support staff	(b)	0.1278***	[0.1169; 0.1387]	− 0.0852***	[-0.0943; − 0.0761]	− 0.0426***	[-0.0506; − 0.0345]
Service workers and trade and market salespeople	(b)	0.1231***	[0.1147; 0.1317]	− 0.0658***	[-0.0728; − 0.0589]	− 0.0573***	[-0.0636; − 0.0511]
Farmers and skilled agricultural, livestock, forestry, and fishing workers	(b)	0.2652***	[0.2408; 0.2897]	− 0.2635***	[-0.2879; − 0.2392]	− 0.0017	[-0.0175: − 0.0141]
Craftsmen and artisans	(b)	0.1424	[0.1320; 0.1529]	− 0.0943***	[-0.1031; − 0.0855]	− 0.0481***	[-0.0558; − 0.0403]
Plant, machine, and assembler operators	(b)	− 0.0071	[-0.0180 0.0037]	− 0.0207	[-0.0296; − 0.0117]	0.0278***	[0.0201; 0.0355]
Quintile I	(a)	− 0.0650***	[-0.0777; − 0.0523]	− 0.0557***	[-0.0668; − 0.0447]	0.1207***	[0.1118; 0.1297]
Quintile II	(a)	− 0.1050***	[-0.1137; − 0.0970]	0.0728***	[0.0661; 0.0795]	0.0322***	[0.0260; 0.0385]
Quintile III	(a)	− 0.0629***	[-0.0707; − 0.0552]	0.0240***	[0.0175; 0.0306]	0.0389***	[0.0330; 0.0448]
Quintile IV	(a)	− 0.0723***	[-0.0786; − 0.0662]	0.0394***	[0.0341; 0.0447]	0.0329***	[0.0281; 0.0378]
Quintile V (base)	(a)						
Health insurance system	Private = 1, Public = 0.	0.0987***	[0.0935; 0.1041]	− 0.0864***	[-0.0910; − 0.0819]	− 0.0123***	[-0.0164; − 0.0082]
Supplementary health insurance	(b)	0.0047	[-0.0010; 0.0105]	0.0380***	[0.0331; 0.0428]	− 0.0427***	[-0.0474; − 0.0381]

Note: dy/dx for factor levels is the discrete change from the base level, which is the margin effect

(a) Inside the range = 1, outside the range = 0; (b) Yes = 1, no = 0

**p < .01; ***p < .001

People whose occupations were classified as “farmers and skilled agricultural, livestock, forestry, and fishing workers” and “managers and administrative directors” had higher AMEs of not accessing the GES plan due to “trust in physician/facility” (26.5% and 16.9%, respectively). Moreover, people whose occupations were related to “craftsmen and artisans” and “administrative support staff” had lower AMEs of not accessing the plan due to waiting lists (-9.4% and − 8.5%, respectively). On the other hand, people whose trades were related to “plant, machine, and assembler operators” were the only ones who presented higher AMEs of not accessing the GES plan due to “lack of information” (2.7%).

People of quintile II had the lowest AMEs of not accessing the GES plan due to “trust in physician/facility” (-10.5%) having quintile V as the base. However, they also provided the highest AMEs of not accessing the GES due to waiting lists (7.2%). In this line, people belonging to the lowest income quintile (quintile I) showed the highest AMEs (12.7%) of not accessing the GES plan to a “lack of information” concerning the highest income quintile (quintile V).

People with private health insurance had higher AMEs (9.8%) of not accessing GES due to “trust in physician/facility” and lower AMEs of not accessing the plan because they “decided not to wait” and “lack of information” (-8.6% and − 1.2%, respectively), regarding people whit public health insurance. At the same time, people having supplementary health insurance had higher AMEs (3.8%) of not accessing GES because they “decided not to wait.“

Regarding health variables (Table 3), people affected by cervical cancer, moderate or severe bronchial asthma, primary arterial hypertension, diabetes mellitus, or depression had the highest AMEs of not accessing the GES plan due to “trust in physician/facility” (24.9%, 13.6%, 13.9%, 13.3%, 8.7%, and 7.6%, respectively). At the same time, people with chronic obstructive pulmonary disease showed the highest AMEs of not accessing Plan GES because they “decided not to wait” (11.1%). Likewise, people with moderate or severe depression and bronchial asthma presented the highest AMEs of not accessing the GES due to “lack of information” (1.8% and 0.5%, respectively).

**Table 3:** Average Marginal Effects (AMEs) of the non-use of the GES health plan, health variables

Variable	Values	Predict (Trust in physician/facility)		Predict (Decided not to wait)		Predict (Lack of information)	
		dy/dx	[95%. Conf. Interv.]	dy/dx	[95%. Conf. Interv.]	dy/dx	[95%. Conf. Interv.]
Primary arterial hypertension	(a)	0.1334***	[0.1259; 0.1411]	− 0.0841***	[-0.0904; − 0.0779]	− 0.0493***	[-0.0552; − 0.0434]
Diabetes mellitus	(a)	0.0876 ***	[0791; 0.0962]	− 0.0717***	[-0.0787; − 0.0647]	− 0.0159***	[-0.0224; − 0.0094]
Depression	(a)	0.0769***	[0684; 0.0854]	− 0.0825***	[-0.0896; − 0.0756]	0.0056***	[-0.0068; 0.01207]
Moderate or severe bronchial asthma	(a)	0.1360***	[0.1242; 0.1479]	− 0.1546***	[-0.1652; − 0.1442]	0.0186***	[0.0100; 0.0272]
Cervical cancer	(a)	0.2493***	[0.2210; 0.2762]	− 0.0886***	[-0.1102; − 0.0669]	− 0.1607***	[-0.1871; − 0.1342]
Chronic obstructive pulmonary disease	(a)	− 0.0756*	[-0.1010; − 0.0502]	0.1101***	[0.0912; 0.1290]	− 0.0345***	[-0.0534; − 0.0157]

Note: dy/dx for factor levels is the discrete change from the base level, which is the margin effect

(a) Yes = 1, no = 0

*p < .05; ***p < .001

## Discussion

Although there are many reasons why people do not use the GES plan, in this study, we consider the three most frequent reasons given by the CASEN 2020 participants, which represented 74% of the total cases (excluding the COVID-19 reason). The first and most frequent reason is “you preferred to choose another physician or facility or to continue with your usual physician” (47%). This explanation alludes to a trust problem that emerges after a first evaluation and derivation from the treating physician. The second alternative, “you decided not to wait to access care through AUGE-GES to solve your problem more quickly,“ implies that the beneficiary opted for faster care outside the GES system. Both explanations suggest that these beneficiaries may choose and take an option other than that offered by the GES system. However, the third explanation points out a lack of information that resulted in the beneficiary not using the GES plan (“I did not know that AUGE-GES covered my condition “; 13%). Following, we will emphasize those results that present AMEs equal to or higher than 5%, considering the grouping indicated in the introduction of this work.

First, regarding demographic variables, women do not access the GES Plan because they are unwilling to wait. Globally, evidence indicates that women are major healthcare users [24], so this reason could underlie the fact that in Chile, women are the ones who decide not to wait. In addition, globally and particularly in Latin America, women are strongly affected by health inequities [24, 25]. These antecedents are consistent with the fact that in Chile, the highest percentage of people on GES waiting lists are women. Among the pathologies with delays are those that mainly affect them (for example, cervical and breast cancer), a worsened situation due to pandemic effects [26].

Likewise, those aged 18 to 35, 36 to 60, and 61years and over are more likely to opt for another physician/facility compared to those under 18, which is not surprising given that these age ranges contain the majority of the population and that they also have decision-making independence and financial autonomy. Likewise, if a person has an indigenous background, it is more likely that they will not have access to GES and will opt for a faster alternative. This piece of information is consistent with the facts that these groups are less favored in public health programs [20, 22] and that in Chile, the largest indigenous population is in the Araucanía region [27], which is one of the regions with the most extended GES waiting lists [28]. As for the migrant population, they are more likely not to access the GES both because of trust in the physician/facility and because of lack of knowledge or lack of information, consistent with previous findings that indicate that this group is disadvantaged in terms of health programs [10, 20]. Critical to understanding this dichotomy, which, on the one hand, indicates that there is a capacity for saving or indebtedness to choose and, on the other hand, suggests a lack of information, is to understand the structural inequalities in Latin America [12, 13].

In terms of geographic areas, the metropolitan region concentrates close to 50% of the country’s population and is, therefore, the region with the most significant provision of health services; thus, it is not surprising that it is the region where there is the greatest probability of opting for alternative care due to distrust in the physician/facility, as indicated by the GES (taking the Austral zone as a base). Likewise, all geographic zones (excluding the Metropolitan Region) have a higher chance of not accessing the GES due to waiting times compared to the Austral zone as a base, which is consistent with the fact that the health services of the Austral zone (Aysén and Magallanes Regions) together present only 1.8% of the cases of delay at the national level by 2020. At the same time, the highest number of days of delay is found in the Central Zone (Valparaíso, O’Higgins, and Maule Regions), whereas the highest average number of waiting days is observed in the Ñuble Region (Southern Zone) [25].

Regarding social variables, people with the private health insurance system (ISAPREs) have the most significant possibility of not having access due to their trust in the physician or the treating institution. This situation is related to the fact that those with private health insurance have the highest income in the country and, therefore, a more significant financial freedom to choose. People in income quintile II (income of approximately US$ 400 per month) are more likely than those in quintile V (US$ 1,500 per month on average) to not have access to the GES program due to the waiting list. This information could be related to the fact that people in Quintile II correspond to those people enrolled in public health insurance at level B (Fondo Nacional de Salud, FONASA-Level B), which is where 60% of the waiting list is concentrated (among the four FONASA groups) [29]. On the other hand, unlike Quintile V (taken as the base), the other lower-income quintiles show that people do not access the GES due to a lack of information or lack of knowledge, a situation that is much more notorious in Quintile I (the lowest income quintile), where it reaches AMEs of 12%. In this line, the adverse effect on health care due to the relationship between health illiteracy and people with lower incomes has been observed previously [30].

People with no formal education are those who access GES the least, seeking faster care due to waiting lists. Although these people are highly likely to have low incomes, they also correspond to the profile of public health insurance users (FONASA A and B), who account for 74% of the delays in the GES program. Regarding occupational categories, there is a greater possibility of not accessing GES due to perceived trust in health professionals or teams, in decreasing order, among “farmers and skilled agricultural, forestry, and fishing workers,“ “managing and administrative directors,“ “professionals, scientists, and intellectuals, " craftsmen and artisans,“ “administrative support staff,“ “service workers and trade and market salespeople,“ “technicians and middle professionals” (all of them with AMEs greater than 10% compared to those who do not belong to those groups). This information suggests that managers, professionals, and skilled workers may have the financial resources (savings or debt) to opt for health care that gives them more confidence.

Finally, regarding health variables, our results indicate that people who have “cervical cancer,“ “bronchial asthma,“ “primary arterial hypertension,“ “diabetes mellitus,“ and “depression” are more likely not to use the GES due to trust in the physician/facility. Moreover, those with “chronic obstructive pulmonary disease” do so because they seek a faster health care service. As of December 2020, the waiting list represented 51,894 people, with an average number of days of delay of 132. In addition, cancer is the health problem with the highest cumulative attention. For example, cervical cancer stands out with 853 persons on the waiting list and an average of 84 days of delay because the oncology units’ operations (both public and private) reduced their capacity due to the conversion of beds for the hospitalization of COVID-19 patients. Persons with chronic obstructive pulmonary disease had a delay of 142 days. Along the same lines, people suffering from depression had a delay of 97 days. In addition, according to official figures, diabetes patients had a delay of 114 days, and those with bronchial asthma of 160 days, those with arterial hypertension of 122 days [25]. All these together indicate that waiting times trigger a particular sensitivity among beneficiaries to seek faster, more high-quality health services.

## Conclusions

Coinciding with the implementation of the health policy known as GES, the quality of the Chilean population’s health has improved [17, 18]. Furthermore, 84% of the beneficiary population uses this mechanism, which indicates high adherence [15], whereas only 16% of the beneficiary population does not use this public policy. The two main reasons for not using the GES are due to trust in the physician/facility and to obtain faster care. Although these two groups of users generate a positive externality by freeing up care quotas and helping to decompress demand, it should be noted that universal access programs seek to provide quality and in-time care to everyone [1–4]. Thus, attracting these beneficiaries seems reasonable if the objective is to implement a program of universal access for all, which is efficient, high-quality, and on time.

Among the profiles of people who opt for health care other than that offered by the GES are those who do so because of the trust in the treating physician or the respective institution (quality). This group generally includes adults with higher incomes (who have greater economic freedom), including part of the migrant population. Coincidently, people who belong to the private health insurance system (with higher incomes), who have a skilled job or occupation that allows them greater financial freedom (availability of savings or debt capacity), people who live in the metropolitan region (those with greater access to health infrastructure), and people who suffer from some pathologies with a greater length of time to receive care. To improve the GES system for this type of beneficiary, together with improvements in the health supply (quality and reduction of waiting lists), health promotion communication campaigns should address the advantages of the GES plan and the achievements obtained through the implementation of this public health policy.

Among the people who do not use the GES program to seek more expeditious care, our findings point out they are mainly people from vulnerable groups: women, people from native ethnic groups, lower-middle income people, those who are in the segment with the most prolonged delay in care (FONASA-Level B), people who do not live in the metropolitan region, and people affected by pathologies with delays in care. Concerning this type of beneficiaries, the GES system must unequivocally improve the timeliness of the health supply (infrastructure, qualified human resources, among others), considering that the current major problems are the delay times or waiting lists [16].

People who do not access GES due to lacking information or knowledge also belong to vulnerable groups: part of the migrant population and people with the lowest economic income [5, 10, 20, 22]. In these cases, the GES program should reinforce the availability of information for these people to make them aware of the program and their rights, improving their health literacy.

In sum, our findings indicate that recipients who do not access GES because of a lack of trust in the physician/facility are predominantly people from the highest income quintiles and have private health insurance (ISAPRES). In turn, those who do not access because they either decided not to wait or lacked information correspond to groups of greater vulnerability. Due to a lack of information, recipients without access reach mainly migrants and people from the lowest income quintile. Likewise, among those who decide not to wait, women, people of native descent, and people without education stand out. Regarding pathologies, the greater probability of not accessing GES in the case of cervical cancer, primary arterial hypertension, and diabetes mellitus is related to trust in the physician/facility, in the case of depression and moderate or severe bronchial asthma due to confidence in the physician/facility and lack of information, while chronic obstructive pulmonary disease due to not waiting. This information reinforces the challenge of refining the model by focusing on managing waiting times rather than referrals and using patient-centered evaluations, especially in those most vulnerable groups that do not access GES because they choose not to wait or lack the necessary information.

As a strength, based on secondary data, this study gives feedback regarding implementing a public policy. It generates information that can contribute to building options to overcome barriers to access to health care, especially for the most vulnerable groups. Regarding limitations, the data used in the study, Casen 2020, were collected in the context of a pandemic. About possible future research, the GES plan could be a suitable basement for a Universal Health Plan. Therefore, it is relevant to continue the follow-up, especially looking for evidence of the interaction between social determinants; beyond each one, looking for the multisectoral and transdisciplinary nature of access to health services.

## Data Availability

The CASEN 2020 database analyzed during the current study is available on the MINDES webpage, http://observatorio.ministeriodesarrollosocial.gob.cl/encuesta-casen-en-pandemia-2020.

## Declarations

The implementing agency of the CASEN 2020 Survey was the Surveys and Longitudinal Studies Center, Catholic University of Chile (PUC). All participants voluntarily participated.

### Authors’ information

SA: Associate professor, Faculty of Business and Economics, University of Talca (Chile).

HV: Lecturer, Psychology Department, University of Talca (Chile).
